# MHC Intratumoral Heterogeneity May Predict Cancer Progression and Response to Immunotherapy

**DOI:** 10.3389/fimmu.2018.00102

**Published:** 2018-01-29

**Authors:** Irene Romero, Cristina Garrido, Ignacio Algarra, Virginia Chamorro, Antonia Collado, Federico Garrido, Angel M. Garcia-Lora

**Affiliations:** ^1^UGC Laboratorios, Complejo Hospitalario de Jaén, Jaén, Spain; ^2^Departamento de Bioquímica, Biología Molecular e Inmunología III, Universidad de Granada, Granada, Spain; ^3^Departamento de Ciencias de la Salud, Universidad de Jaén, Jaén, Spain; ^4^Servicio de Análisis Clínicos e Inmunología, UGC Laboratorio Clínico, Hospital Universitario Virgen de las Nieves, Instituto de Investigación Biosanitaria ibs.Granada, Granada, Spain; ^5^Unidad de Biobanco, Hospital Universitario Virgen de las Nieves, Instituto de Investigación Biosanitaria ibs.Granada, Granada, Spain

**Keywords:** intratumoral heterogeneity, MHC, metastases, T lymphocytes, immunotherapy

## Abstract

An individual tumor can present intratumoral phenotypic heterogeneity, containing tumor cells with different phenotypes that do not present irreversible genetic alterations. We have developed a mouse cancer model, named GR9, derived from a methylcholanthrene-induced fibrosarcoma that was adapted to tissue culture and cloned into different tumor cell lines. The clones showed diverse MHC-I phenotypes, ranging from highly positive to weakly positive MHC-I expression. These MHC-I alterations are due to reversible molecular mechanisms, because surface MHC-I could be recovered by IFN-γ treatment. Cell clones with high MHC-I expression demonstrated low local oncogenicity and high spontaneous metastatic capacity, whereas MHC-I-low clones showed high local oncogenicity and no spontaneous metastatic capacity. Although MHC-I-low clones did not metastasize, they produced MHC-I-positive dormant micrometastases controlled by the host immune system, i.e., in a state of immunodormancy. The metastatic capacity of each clone was directly correlated with the host T-cell subpopulations; thus, a strong decrease in cytotoxic and helper T lymphocytes was observed in mice with numerous metastases derived from MHC-I positive tumor clones but a strong increase was observed in those with dormant micrometastases. Immunotherapy was administered to the hosts after excision of the primary tumor, producing a recovery in their immune status and leading to the complete eradication of overt spontaneous metastases or their decrease. According to these findings, the combination of MHC-I surface expression in primary tumor and metastases with host T-cell subsets may be a decisive indicator of the clinical outcome and response to immunotherapy in metastatic disease, allowing the identification of responders to this approach.

## Introduction

It is widely accepted that tumors can display intratumoral genetic heterogeneity, and attention has recently focused on its implications for clinical outcomes, metastatic progression, and the response to different therapies (1–4). Hence, the clinical practice of ordering a single biopsy from a progressing tumor may not be adequate to assess intratumoral complexity and, importantly, may provide an incomplete view of potential therapeutic targets. Most therapeutic decisions are based on the analysis of primary tumors, which can often be successfully removed, with metastatic outgrowth being largely responsible for the death of patients. Three types of heterogeneity should, therefore, be considered relevant in tumorigenesis: intratumoral, intrametastatic, and intermetastatic (5, 6).

Intratumoral heterogeneity involves not only genetic alterations but also epigenetic mechanisms and/or differences in protein expression levels in response to microenvironment signals or post-transcriptional modifications. The immunological sculpting of tumor lesions can also influence intratumoral cell variability. Thus, immunoediting removes the more immunogenic tumor cells in a primary tumor, whereas different immune escape mechanisms are developed by less immunogenic cells (7). One well-documented method by which tumor cells avoid immune surveillance is MHC-I downregulation, which renders tumor cells invisible to T-cell-mediated cytotoxicity (immunoblindness) (8–10).

We previously reported that MHC-I molecules may act as tumor suppressor genes (8, 11). In human melanoma cell lines, loss of MHC-I expression promoted higher oncogenicity *in vitro* and *in vivo* in immunodeficient mice. Moreover, transfection of one lost HLA class I allele reverted these oncogenic properties. In preclinical cancer models, tumors or tumor cell lines are usually considered to be composed of identical cells and are studied as a single cell. The present study offers a novel analysis of intratumoral MHC-I heterogeneity in the GR9 tumor mouse model, which comprises a set of tumor clones with different H-2 class I surface expressions that exhibit a wide range of MHC-I phenotypes. It was previously observed that MHC-I expression is restored on all tumor clones by IFN-γ treatment, demonstrating that these MHC-I alterations are reversible defects (soft lesions) (12). Hence, the GR9 mouse tumor model is highly suitable for confirming results obtained in human tumors. We hypothesized that intratumor heterogeneity in MHC-I surface expression and MHC-I expression on metastases may determine the tumor progression and response to anti-metastatic immunotherapy. For this purpose, spontaneous metastatic assays were performed that corresponded to the clinical phases of primary tumor detection, surgery, and therapeutic intervention. It is possible that the combination of the MHC-I surface expression of primary tumor and metastases along with host T-cell subsets could play a decisive role as a biological marker of the clinical outcome and response to immunotherapy in patients with metastatic progression.

## Materials and Methods

### Cell Lines and Stimulation

The GR9 cell line is derived from a mouse fibrosarcoma induced by methylcholanthrene in BALB/c mice and has been extensively characterized in our laboratory. It is composed of cell clones with different H-2 class I expression patterns (13). Spontaneous metastasis assays were performed with four GR9 cell clones A7, B7, C5, and B11, which were obtained by limited dilution method from the GR9 cell line and were recloned by capturing individual cells under phase contrast microscopy. All cell lines were characterized by PCR assay using short tandem repeats and were regularly tested for MHC-I surface expression. Cell lines were maintained in Dulbecco’s medium (Sigma-Aldrich) supplemented with 10% fetal bovine serum (Life Technologies), 2 mM glutamine, and antibiotics. In some experiments, cell lines were treated with 100 U/ml IFN-γ for 48 h (Sigma-Aldrich) or with 500 nM trichostatin A (TSA) for 48 h to inhibit histone deacetylase (HDAC) activity.

### Animals

Eight-week-old male BALB/c mice (Charles River Laboratories) were used in experiments. This study was carried out in accordance with the recommendations of European Community Directive 86/609/CEE and Spanish law (Real Decreto 1201/2005) on the use of laboratory animals, and their housing and the experimental procedures were approved by the Junta de Andalucía animal care committee and adhered to animal welfare guidelines of the National Committee for Animal Experiments.

### *In Vivo* Local Tumor Growth Assay

In the *in vivo* oncogenicity assays, 6.25 × 10^5^ cells of each cell clone were subcutaneously injected into the footpad in groups of 10 mice. The growth of local tumors was recorded three times/week in all animals, measuring the largest diameter of each tumor with electronic calipers.

### Spontaneous Metastasis Assay

Four different cell doses (50 × 10^5^, 25 × 10^5^, 12.5 × 10^5^, and 6.25 × 10^5^) of each clone were injected into the footpad of syngeneic BALB/c mice. When the growth of local tumors reached 10 mm, they were excised under anesthesia with 0.04 ml diazepam (Valium, Roche) and 0.1 ml ketamine (Ketolar, Pfizer). The primary tumors were removed with sterilized instruments, using electrocautery to minimize bleeding and closing the wound with surgical clips and adhesive. After the surgery, each animal was housed alone until recovery from anesthesia. When signs of disease were observed, the animals were anesthetized and euthanized by cervical dislocation, followed by complete necropsy and count of metastases. Local tumors and macroscopically visible metastatic nodules were excised, disaggregated, and adapted to tissue culture. Next, lungs of the mice were fixed in Bouin’s solution for a micrometastasis count.

### Experimental Metastasis Assay

Doses of 2.5 × 10^5^ cells of B11 or A7 cell clones in 0.2 ml of PBS were injected into the lateral tail vein in groups of 10 syngeneic BALB/c mice. On day 30 postinjection, animals were anesthetized and euthanized by cervical dislocation. Complete necropsy was done, as described above.

### MHC Class I Expression

MHC class I expression was analyzed by indirect immunofluorescence using FACS (FACScan; Becton Dickinson) according to a standard protocol. Briefly, 5 × 10^5^ cells were washed twice with PBS and incubated for 30 min at 4°C with the primary antibodies anti-H-2 K^d^ (K9.18), anti-H-2 D^d^ (34.5.8), and anti H-2 L^d^ (28.14.8 and 30.5.7), all obtained from the ATCC (Rockville, MD, USA). The secondary fluorescein isothiocyanate (FITC)-conjugate antibody (anti-mouse FITC IgG/Fab, Sigma-Aldrich) was used at 1:120 dilution for 30 min at 4°C in the dark. Isotype-matched non-immune mouse IgG and cells labeled with the fluorescein-conjugated antibody alone served as controls. A minimum of 1 × 10^4^ cells were analyzed with CellQuest-Pro software. All cell lines were studied in baseline conditions and after IFN-γ treatment.

### Immunotherapy, Chemotherapy, and Chemoimmunotherapy Protocols

Each of the following four treatment protocols was applied in groups of 20 mice:

1. Weekly intraperitoneal (i.p.) administration of protein-bound polysaccharide K (PSK) at 2.5 mg/mouse/week in 500 µl of saline solution. PSK is derived from the fungus *Coriolus versicolor* and has demonstrated anticancer activity in experimental models and in clinical trials (14, 15), with results suggesting that PSK mainly acts as an immunomodulator of biological response. PSK is prepared by extracting cultured mycelia of *C. versicolor* with hot water; next, the precipitate is separated from the clear supernatant with saturated ammonium sulfate and then desalted and dried. PSK, kindly provided by *Kureha Chemical Ind. Co. (Tokyo, Japan)*, was dissolved in saline solution and heated at 50°C for 30 min until the appearance of a clear solution, which was then filtered by 0.22 µm filters (Millipore, Spain).
2. Weekly i.p. injection of CpG ODN 1826 and irradiated autologous tumor cells (CpG+IR−TC): 20 µg of CpG ODN 1826 (InvivoGen, San Diego, CA, USA) were resuspended in 200 µl saline solution; while one million autologous tumor cells collected in Dulbecco’s medium and irradiated with a dose of 100 Gy were maintained in culture for 48 h, washed with PBS, and resuspended in 200 µl saline solution. Both solutions were administered to each mouse in this group.
3. Weekly i.p. injection of docetaxel (Taxotere, 80 mg/2 ml, Sanofi Aventis, Barcelona, Spain) diluted to 10 mg/ml according to the manufacturer’s instructions and further diluted to 625 µg/ml in sterile saline solution, administering 125 μg/mouse/week.
4. Weekly i.p. injection of PSK plus docetaxel: mice were simultaneously i.p. injected with PSK and docetaxel.
5. These weekly injections were administered during a 6-week period from day 7 after local tumor excision (Figure 4A). In each treatment group, 10 mice were euthanized at 1 week after the end of the treatment (on day 50 after primary tumor extirpation), while the remaining 10 mice were euthanized on day 100 after primary tumor extirpation, except that all mice in the docetaxel group were euthanized on day 50 due to signs of disease. In control groups, 10 tumor cell-injected mice receiving a weekly i.p. injection of 200 µl of saline solution were all euthanized on day 50 after primary tumor extirpation. The same protocols were applied to tumor-free animals in order to evaluate the toxicity of the treatments and their effect on the survival rate.

### Preparation of Splenic and Lung-infiltrating Leukocytes

Spleens and lungs were excised and gently homogenized in a Stomacher blender in cold PBS (Sigma-Aldrich). A tissue fragment was removed, and a sterile falcon cell strainer (BD Bioscience, Madrid, Spain) was used to create a single-cell suspension. Red blood cells were lysed with ACK lysing buffer (Gibco, Paisley, UK) for 5 min and then washed twice in PBS. Viable cells were counted and used for the antibody staining reaction.

### Flow Cytometry Analysis of Immune Cell Subsets

The following labeled antibodies (Miltenyi Biotec, GmbH, Germany) were used for the direct immunofluorescence study: CD45-PE, CD3*_ε_*-APC, CD4-FITC, CD8-PE, CD25PE, FoxP3-APC, CD19-FITC, and CD49b-FITC. Isotype-matched non-immune mouse IgGs conjugated with FITC, PE, or APC, and unstained cells served as controls. FcR Blocking Reagent was used to block unwanted binding of antibodies to mouse cells expressing Fc receptors. Briefly, 5 × 10^5^ cells were washed twice with PBS and incubated for 10 min at 4°C in the dark with the primary antibodies. For the determination of T regulatory (Treg) cells, cells were incubated for 10 min at 4°C in the dark with anti-CD4 and anti-CD25 antibodies after permeabilization for 30 min and incubation for 30 min at 4 °C with anti-FoxP3 antibody. The percentage of T-, B-, NK-, and NKT-lymphocytes was determined with respect to total lymphocytes. The percentage of CD4+T lymphocytes and CD8+T lymphocytes was determined with respect to CD3+T lymphocytes, and the percentage of Treg cells with respect to CD4+T lymphocytes. Cells were analyzed on a FACSCanto cytometer (BD Bioscience). Each sample contained at least 5 × 10^4^ cells and was analyzed with CellQuest-Pro software.

### RT and Quantitative Real-time PCR

Quantitative RT-PCR was performed as previously described (16). An mRNA isolation kit (Miltenyi-Biotech) was used to extract mRNA from tumor cell lines. First-strand cDNA was synthesized with 100 ng mRNA using a High Capacity Reverse Transcription Kit (Applied Biosystems, Foster City, CA, USA) in a total volume of 20 µl. These cDNAs were diluted to a final volume of 100 µl. Real-time quantitative PCR analyses were performed in the 7500 Fast System (Applied Biosystems), performing PCR reactions in quadruplicate and expressing the values obtained as means ± SD. Quantitative PCR was performed with the Power SYBR Green Master mix (Applied Biosystems) using previously reported primers and amplicon size for each gene, with GADPH and β-actin genes serving as housekeeping genes. PCR conditions were 40 cycles of 15 sec of denaturation at 95°C and 60 s at 60°C.

### Statistical Analysis

Prism Software (Graph Pad Software V5.0) and SPSS Statistical 17.0 (IBM SPSS Inc., Chicago, IL, USA) were used for statistical analyses. A two-tailed unpaired Student’s *t*-test or Mann–Whitney *U* test was used for statistical comparisons between two groups. ANOVA followed by the Tukey *post hoc* analysis or Kruskal–Wallis test followed by the Dunn’s post-test was used for multiple comparisons. All data are expressed as the mean ± SD. A *p* value less than 0.05 was considered statistically significant (*p < 0.05, **p < 0.01).

## Results

### H-2 Class I Phenotypes of GR9 Fibrosarcoma Clones

**Figure 1:**
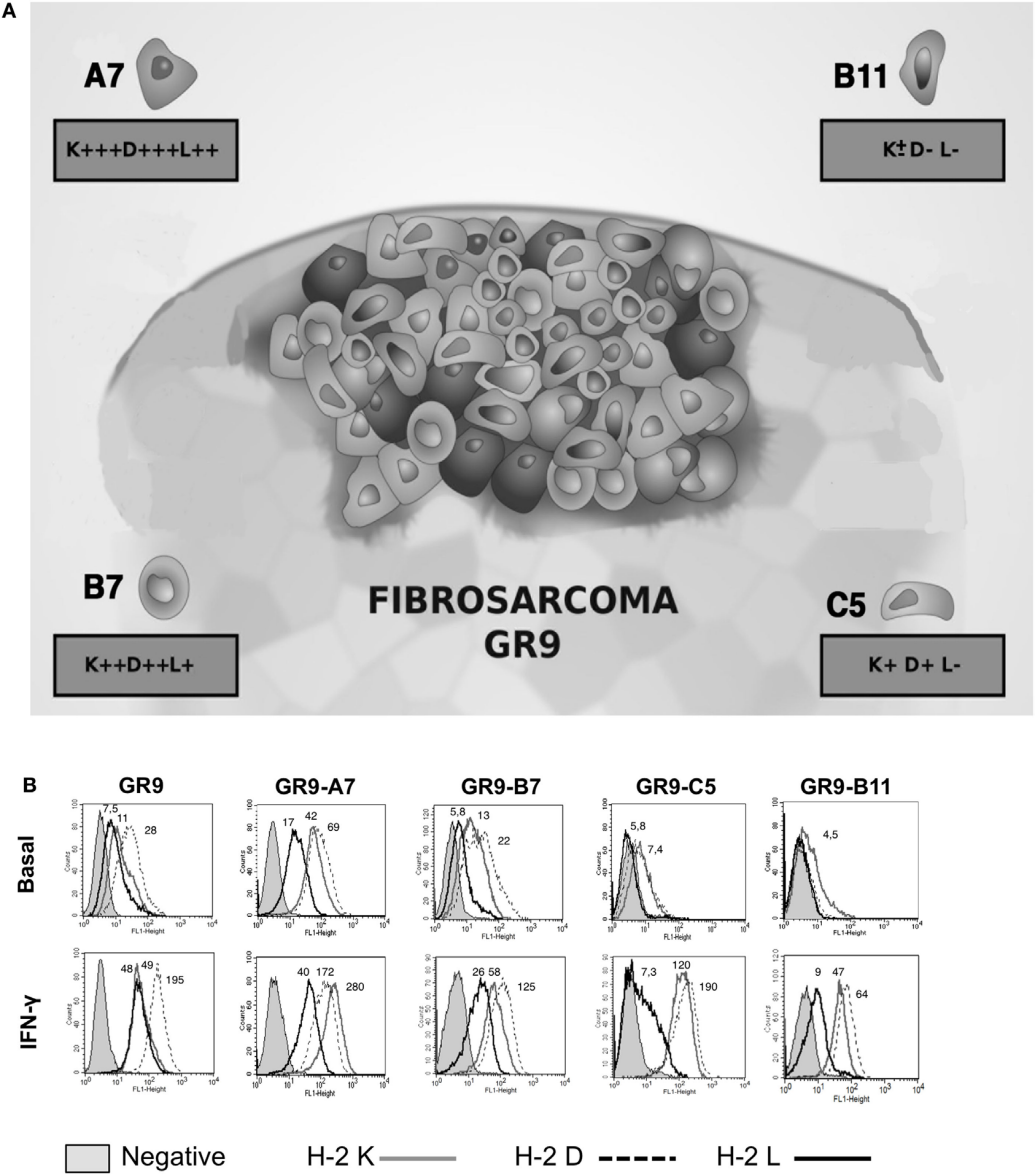
**(A)** MHC-I intratumoral heterogeneity in MCA-induced fibrosarcoma GR9; **(B)** H-2 class I surface expression of GR9, GR9-A7, GR9-B7, GR9-C5, and GR9-B11 clone cell lines in baseline conditions and after treatment with IFN-γ (100 U/ml) for 48 h: H-2 K^d^ (*gray line)*, H-2 D^d^ *(dotted line)*, and *H-2* L^d^ (*black line)*. GR9-A7 showed high levels of H-2 class I expression, GR9 and GR9-B7 showed intermediate levels, and GR9-C5 low levels, while GR9-B11 is practically negative in baseline conditions; all three molecules were induced after IFN-γ treatment in all GR9 clones. Data from one experiment are depicted.

Analysis of MHC-I surface expression in the GR9 tumor clone cell lines revealed diverse MHC-I phenotypes ranging from MHC-I negative to MHC-I positive. Clones with high (GR9-A7), intermediate (GR9-B7), low (GR9-C5), and very low (very weak expression of only one locus) (GR9-B11) MHC-I phenotypes were selected to represent intratumoral heterogeneity (Figure 1A). The GR9 cell line exhibits the cell surface expression of the H-2 K^d^, D^d^, and L^d^ molecules, and all three can be induced by IFN-γ treatment (Figure 1B). In the GR9-A7 clone, expressions of H-2 K^d^, D^d^, and L^d^ molecules were positive under baseline conditions; in the GR9-B7 clone, the expression of all three molecules was positive under baseline conditions but levels were lower than in the GR9-A7 clone; in the GR9-C5 clone, expression of H-2 K^d^ and D^d^ molecules was very low under baseline conditions and there was no expression of H-2 L^d^ molecule; finally, in the GR9-B11 clone, H-2 K^d^ expression was very weak and there was no expression of D^d^ or L^d^ molecules (Figure 1B). In all GR9 clones, the expression of all three H-2 class I molecules was induced after treatment with IFN-γ (Figure 1B), indicating the absence of structural defects or “hard lesions.”

### Molecular Mechanisms Involved in MHC Class I Downregulation

**Figure 2:**
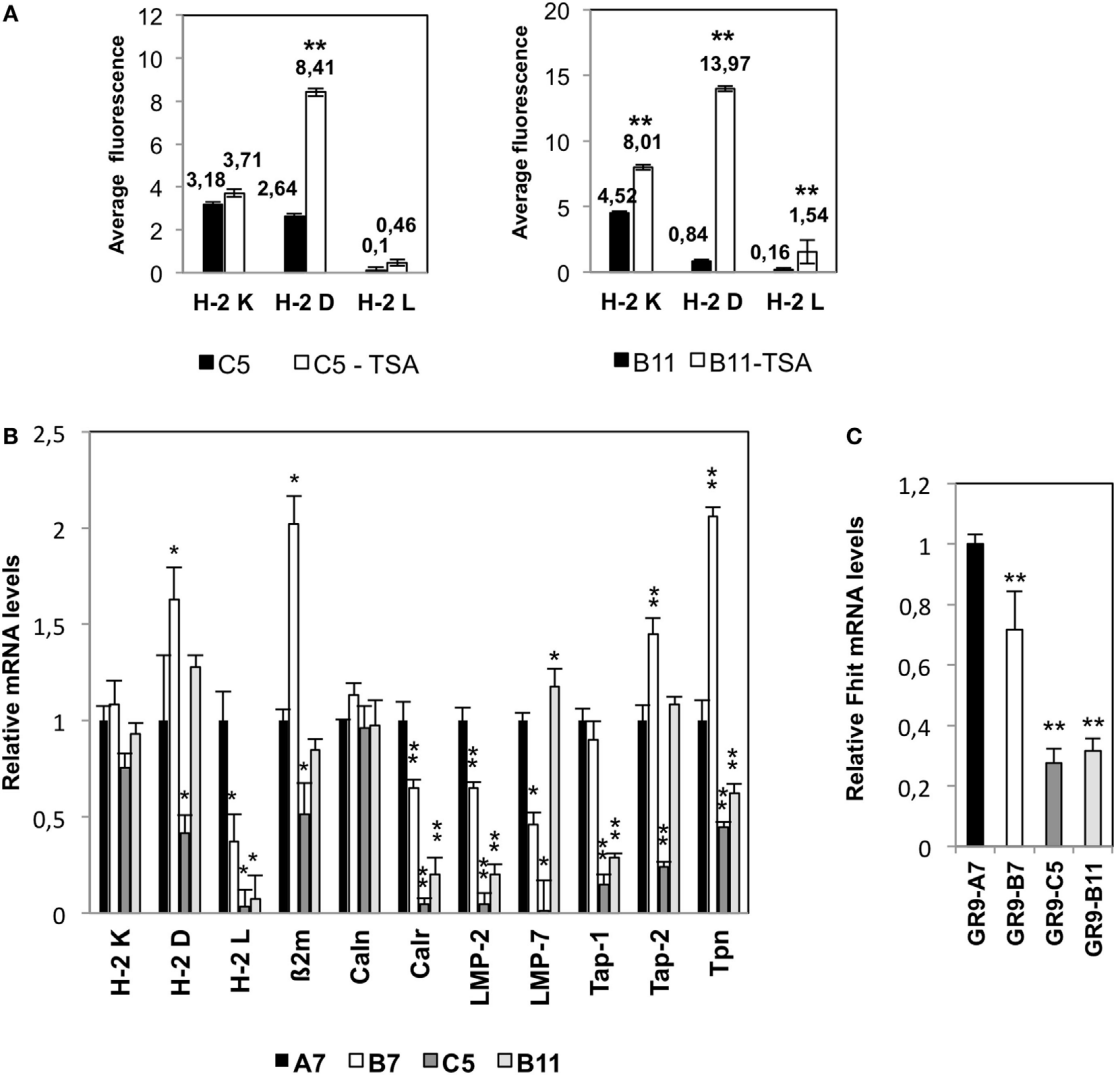
**(A)** Mean fluorescence values for H-2 class I expression under baseline conditions and after treatment with trichostatin A (TSA, 500 nM for 48 h). A representative example of three independent experiments is depicted. **p < 0.01. A two-tailed Student *t*-test was used for statistical analysis. **(B)** Transcription levels of H-2 class I heavy chain, β_2_-microglobulin, and several APM components detected by real-time RT-PCR. **(C)** Expression of Fhit in fibrosarcoma cell lines. Expression levels of the genes of interest were determined relative to levels of β-actin and GADPH housekeeping genes. Data for GR9-A7 are set at 1. Values are depicted as means ± SD of three independent experiments performed in quadruplicate. *p < 0.05; **p < 0.01 vs. the A7 values. ANOVA test, followed by Tukey *post hoc* test was used for statistical analysis.

In order to determine whether HDAC is implicated in the regulation of H-2 class I surface expression, GR9-C5 and -B11 clones were treated with TSA with the following results: in the GR9-C5 clone, TSA treatment produced a threefold increase in H-2 D^d^ molecule expression and a slight increase in H-2 K^d^ and L^d^ molecules; in the GR9-B11 clone, TSA treatment produced an increase of more than 16-fold in H-2 D^d^ molecule expression, 9.6-fold in H-2 L^d^ expression, and 1.8-fold in H-2 K^d^ molecule expression (Figure 2A).

Expression profiles revealed by mRNA next-generation sequencing were highly similar among the four tumor clones, which showed significant graded differences in the transcriptional expression of some genes related to antigen processing and presentation machinery and in the Fhit gene. Other differentially expressed genes showed no such graded differences in expression among the four tumor clones with varied MHC-I surface expressions. We also used flow cytometry to determine the expression of other markers involved in the immune response (PD-1, PD-L1, CD137L, CD137, TIM3, Galectin-9, Fas, and FasL) finding no significant differences among these four tumor clones. The molecular mechanisms underlying the altered H-2 class I phenotypes in the GR9-B7, GR9-C5, and GR9-B11 clones involve the coordinated transcriptional downregulation of several APM components. Analysis by real-time RT-PCR showed that transcriptional expression of calreticulin, LMP-2, and LMP-7 genes was lower in GR9-B7 cells than in GR9-A7 cells. Tap-1, Tap-2, and tapasin were also downregulated in GR9-C5 cells, while calreticulin, LMP-2, Tap-1, and tapasin were downregulated in GR9-B11 cells (Figure 2B).

Our group previously reported the direct involvement of the Fhit tumor suppressor gene in the transcriptional regulation of APM components (16). The mRNA expression of Fhit in the GR9-A7 clone was 1.4-fold higher than in the GR9-B7 clone, around 3.6-fold higher than in the GR9-C5 clone, and 3.3-fold higher than in B11 cells (Figure 2C).

### H-2 Class I Phenotype Influences *In Vivo* Local Tumor Growth and Spontaneous Metastatic Capacity

**Figure 3:**
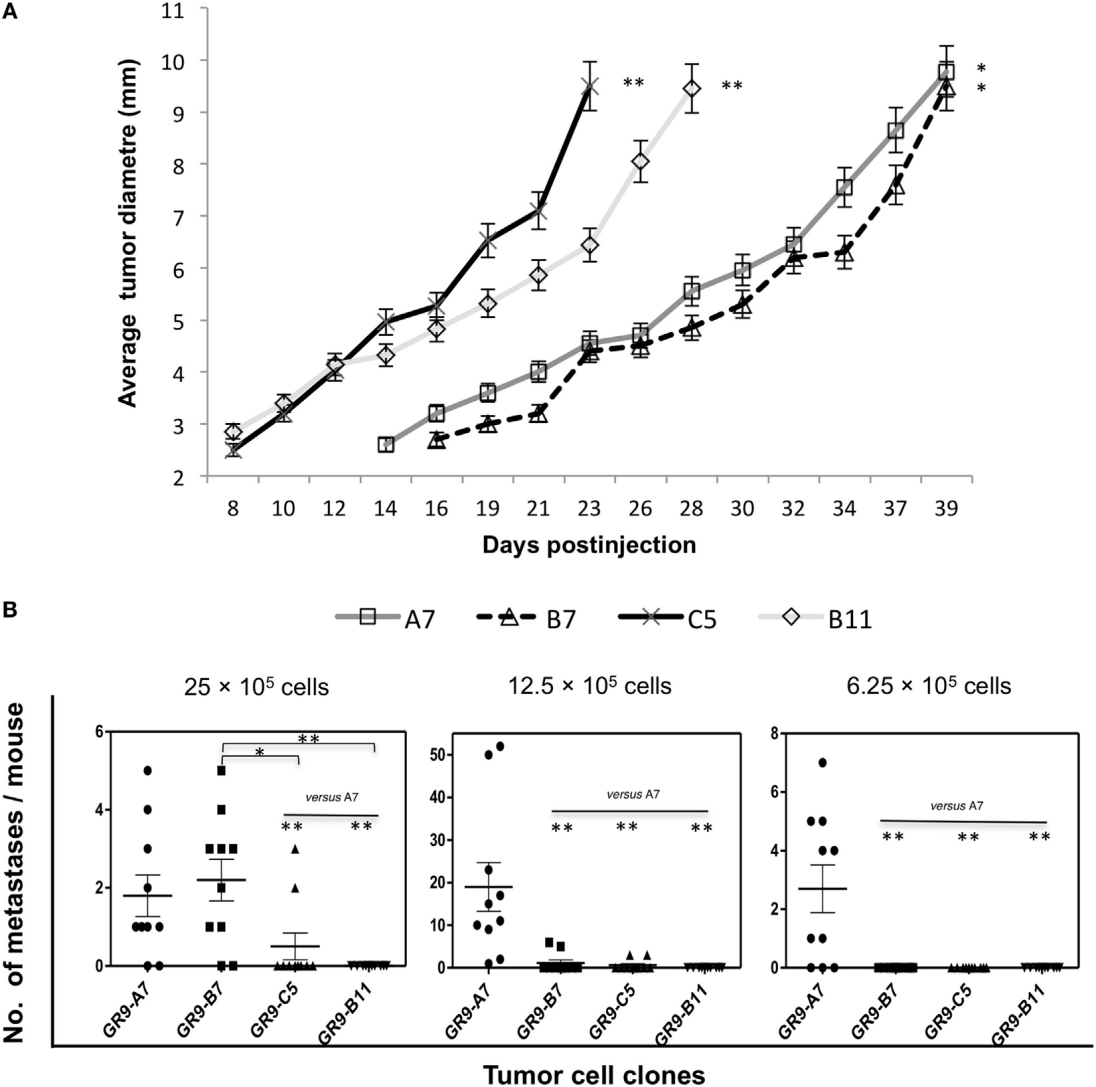
**(A)** Cell lines with different patterns of H-2 class I expression show distinct local oncogenicity. Tumor clones were injected (6.25 × 10^5^ cells) into the footpad of male BALB/c mice. The tumor diameter was measured three times/week and removed when it reached 10 mm. H-2 class I-positive tumors, GR9-A7 and -B7, grew more slowly than the more negative tumors GR9-C5 and -B11. The mean ± SD tumor diameter of 10 mice is shown as a function of time. **(B)** Spontaneous metastasis assays with GR9-A7, -B7, -C5, and -B11 tumor clones. Four different cell doses (50 × 10^5^, 25 × 10^5^, 12.5 × 10^5^, and 6.25 × 10^5^ cells) were injected into the footpad of 10 mice each. The number of metastases per mouse was counted. GR9-B11 clone (MHC-I-negative) did not generate metastases at any tumor cell dose. GR9-A7, -B7, and -C5 clones all generated metastases. GR9-A7 clone (MHC-I-positive) showed the highest metastatic capacity (range, 1–52 metastases per mouse). *p < 0.05; **p < 0.01. Kruskal–Wallis test, followed by Dunn *post hoc* test was used for statistical analysis.

Assays were conducted at different cell doses, using the lowest dose (6.25 × 10^5^ cells) to measure the local growth rate. Local tumors began to grow on day 8 in mice injected with GR9-C5 and -B11 cells and were removed on days 23 and 28, respectively, whereas they began to grow on days 14 and 16 postinjection, respectively, in those injected with GR9-A7 and -B7 cells and were removed on day 39 (Figure 3A).

Spontaneous metastasis assays were conducted to evaluate the *in vivo* metastatic potential. Ninety percent of mice injected with GR9 cells developed 2–9 pulmonary metastases (PM) and 0–1 lymph node metastases (LNM). Each tumor cell clone was then injected at different doses (50 × 10^5^, 25 × 10^5^, 12.5 × 10^5^, and 6.25 × 10^5^ cells). As shown in Figure 3B, the highest spontaneous metastatic capacity was shown by GR9-A7 cells, which generated overt LNMs and PMs (range, 1–52; 70–90% of the mice) at three of the four cell doses. Intermediate metastatic capacity was shown by GR9-B7 cells, which generated PMs (range, 1–6) at a cell dose of 25 × 10^5^ (80% of mice) or 12.5 × 10^5^ cells (20% of mice). A very low metastatic capacity was found for GR9-C5 cells, observing PMs (range, 1–3) in only 20% of animals. No overt metastases were produced at any dose by GR9-B11 (MHC-I-negative) cells, although hosts injected with this clone presented micrometastases in permanent immunodormancy (17). At necropsy, all of the mice injected with the highest dose (50 × 10^5^ cells) were found to be overt metastasis-free, regardless of the tumor cell line used (data not shown). This may be attributable to the rapid growth of the local tumor, which was excised at 12 days after reaching a diameter of 10 mm. As mentioned above, the four tumor clones were classified into four groups according to their MHC-I cell surface expression, from highly positive to weakly positive. We analyzed the oncogenic and metastatic properties of a minimum of two clones per MHC-I group, finding that the results for the two clones were always completely reproducible. According to these *in vivo* assay findings, H-2 class I surface expression may be directly related to the metastatic capacity and inversely related to the local oncogenicity.

### Changes in Immune Cell Subpopulations in Tumor-Bearing Mice

Host immune cell populations were analyzed on day 50 after tumor primary extirpation, when metastatic spread occurred. In lung-infiltrating lymphocytes, in comparison to wild-type mice, GR9 fibrosarcoma-bearing mice exhibited a strong decrease in several immune cell subpopulations, with a significant reduction (*p* < 0.05) in TCD3+ (36.1 vs. 51.9%), TCD8+ (6.5 vs. 9.7%), and TCD4+ (29.6 vs. 40.6%) lymphocytes; moreover, a significant increase in Treg cells was found (8.9 vs. 2.9% of CD4+ cells) (Table 1). Very similar results were also observed in GR9-B7 tumor-bearing mice, with a significant decrease (*p* < 0.05) in TCD3+ (42.3 vs. 51.9%), mainly TCD4+ (35.1 vs. 40.6%), and TCD8+ (7.2 vs. 9.7%) lymphocytes, and a significant increase in Treg (5.8 vs. 2.9% of CD4+) (Table 1). GR9-A7 tumor-bearing mice also showed a slight decrease in several immune cell subsets, with a reduction in TCD3+ lymphocytes vs. wild-type animals (43.3 vs. 51.9%) due to a decrease in TCD8+ (5.9 vs. 9.7%) and TCD4+ (37.4 and 40.6%) subpopulations; while a significant increase in Treg cells was observed (9.9 vs. 2.9% of CD4+). A strong increase in T lymphocytes was observed in GR9-B11 tumor-bearing mice, with a rise in TCD3+ lymphocytes (64.1 vs. 51.9%), due to an increase in T-helper (50.5 vs. 40.6%), and T-cytotoxic (12.1 vs. 9.7%) lymphocytes. Analysis of lymphocyte populations in splenic leukocytes showed very similar changes to those found in lung-infiltrating lymphocytes. In comparison to wild-type mice, GR9 fibrosarcoma-bearing mice exhibited a strong decrease in several immune cell subpopulations, with a significant reduction (*p* < 0.05) in TCD3+ (18.0 vs. 33.1%), TCD8+ (3.4 vs. 6.9%), and TCD4+ (14.6 vs. 26.1%) lymphocytes; moreover, a significant increase in Treg cells was found (10.6 vs. 4.4% of CD4+ cells) (Table 1). Very similar results were also observed in GR9-B7 tumor-bearing mice, with a significant decrease (*p* < 0.05) in TCD3+ (25.3 vs. 33.1%), mainly TCD4+ (19.7 vs. 26.1%), and TCD8+ (5.7 vs. 6.9%) lymphocytes, and a significant increase in Treg cells (6.9 vs. 4.4% of CD4 +) (Table 1). GR9-A7 tumor-bearing mice also showed a slight decrease in several immune cell subsets, with a reduction in TCD3+ lymphocytes vs. wild-type animals (30.3 vs. 33.1%) due to a decrease in TCD8+ (5.8 vs. 6.9%) and TCD4+ (24.4 and 26.1%) subpopulations; while a significant increase in Treg cells was observed (10.8 vs. 4.4% of CD4+). A strong increase in T lymphocytes was observed in GR9-B11 tumor-bearing mice, with a rise in TCD3+ lymphocytes (53.7 vs. 33.3%), due to an increase in T-helper (40.4 vs. 26.1%) and T-cytotoxic (13.0 vs. 6.9%) lymphocytes and a slight decrease in Treg cells (3.6 vs. 4.4%). Analysis of lymphocyte populations in peripheral blood from all mouse groups yielded the same results as observed in the spleen and the lungs (data not shown).

**Table 1:** Changes in lung-infiltrating and splenic lymphocyte subpopulations.

		CD3^+^	CD4^+^	CD8^+^	CD3^+^CD4^+^CD25^+^FoxP3^+a^	CD3^−^CD19^+^	CD3^−^CD49b^+^	CD3^+^CD49b^+^
**Lung lymphocyte subpopulations in tumor-bearing mice and wild-type**								
Wild-type		51.9 ± 6.3	40.6 ± 5.5	9.7 ± 1.5	2.9 ± 0.7	35.2 ± 5.9	12.2 ± 0.9	1.8 ± 0.8
GR9		36.1 ± 4.7*	29.6 ± 2.4*	6.5 ± 0.8*	8.9 ± 2.1*	49.1 ± 4.8*	14.1 ± 0.6*	1.4 ± 0.2
GR9-A7		43.3 ± 4.1*	37.4 ± 3.3	5.9 ± 1.1*	9.8 ± 2.6*	43.0 ± 6.0	12.0 ± 2.0	2.0 ± 0.5
GR9-B7		42.3 ± 5.5*	35.1 ± 4.0*	7.2 ± 1.5*	5.4 ± 1.1*	43.2 ± 4.5	14.0 ± 0.7*	2.4 ± 0.3*
GR9-B11		64.1 ± 5.2*	50.5 ± 6.9*	12.1 ± 2.2*	3.5 ± 0.6	26.2 ± 2.1*	9.6 ± 4.0	1.6 ± 0.3
**Splenic lymphocyte subpopulations in tumor-bearing mice and wild-type**								
Wild-type		33.1 ± 4.3	26.1 ± 3.1	6.9 ± 1.8	4.4 ± 0.8	61.0 ± 4.2	4.6 ± 1.0	0.4 ± 0.3
GR9		18.0 ± 1.6*	14.6 ± 1.2*	3.4 ± 0.5*	10.6 ± 2.5*	72.2 ± 5.8*	9.6 ± 1.6*	0.3 ± 0.2
GR9-A7		30.3 ± 7.0	24.4 ± 5.3	5.8 ± 1.7	10.8 ± 4.2*	65.5 ± 6.0	4.0 ± 2.0	0.9 ± 0.5
GR9-B7		25.3 ± 7.4*	19.7 ± 7.0*	5.7 ± 1.5*	6.9 ± 2.1*	67.0 ± 9.5	7.7 ± 2.0*	1.0 ± 0.4*
GR9-B11		53.7 ± 5.5*	40.4 ± 4.1*	13.0 ± 2.9*	3.6 ± 1.7	41.0 ± 4.7*	3.7 ± 1.0	1.4 ± 0.6*
**Splenic lymphocyte subpopulations in tumor-bearing mice after immuno- or chemoimmunotherapy**								
GR9 Groups	CpG + GR9	19.9 ± 3.1	16.5 ± 2.8	3.3 ± 0.3	11.5 ± 2.9	75.3 ± 13.0*	4.7 ± 0.7*	0.2 ± 0.1*
	PSK	32.1 ± 5.6*	26.0 ± 6.4*	6.2 ± 0.7*	9.1 ± 1.1	61.8 ± 0.6*	5.9 ± 1.6	0.4 ± 0.1
	PSK + Docetaxel	30.8 ± 3.4*	19.7 ± 2.4*	8.5 ± 2.8*	10.1 ± 5.2	60.1 ± 5.1*	8.9 ± 6.3*	0.8 ± 1.1*
GR9-A7 Groups	CpG + A7	46.5 ± 5.3*	31.9 ± 3.8*	14 ± 2.2*	4.7 ± 3.8*	49.5 ± 12.4	3.8 ± 1.3	2.0 ± 0.8
	PSK	58.0 ± 17.5*	44.2 ± 15.2*	13.7 ± 2.4	9.5 ± 3.8*	30.8 ± 15.5*	11.1 ± 0.6*	1.6 ± 0.1
	PSK + Docetaxel	36.6 ± 3.1*	30.1 ± 2.2*	6.5 ± 0.8	1.06 ± 0.3*	60.2 ± 5.2	3.0 ± 0.1	0.9 ± 0.1*
GR9-B7 Groups	CpG + B7	21.6 ± 5.5	16.2 ± 5.4	5.5 ± 0.8	5.6 ± 1.8	73.7 ± 7.4*	4.6 ± 1.7*	0.6 ± 0.3*
	PSK	32.2 ± 8.5	23.0 ± 6.0	7.6 ± 2.9	9.0 ± 2.5*	62.4 ± 8.3	5.3 ± 1.6	0.7 ± 0.3
	PSK + Docetaxel	26.0 ± 7.14	20.6 ± 4.2	5.1 ± 3.2	9.6 ± 3.3*	70.2 ± 5.7*	3.6 ± 1.0*	0.4 ± 0.3*

*Data are expressed as means ± SD*.

*^a^Percentage among CD4+ T cells*.

*p < 0.05 compared with wild-type or untreated tumor-bearing mice.

### Anti-Metastatic Preclinical Assays with Immunotherapy and Chemoimmunotherapy Treatments

**Figure 4:**
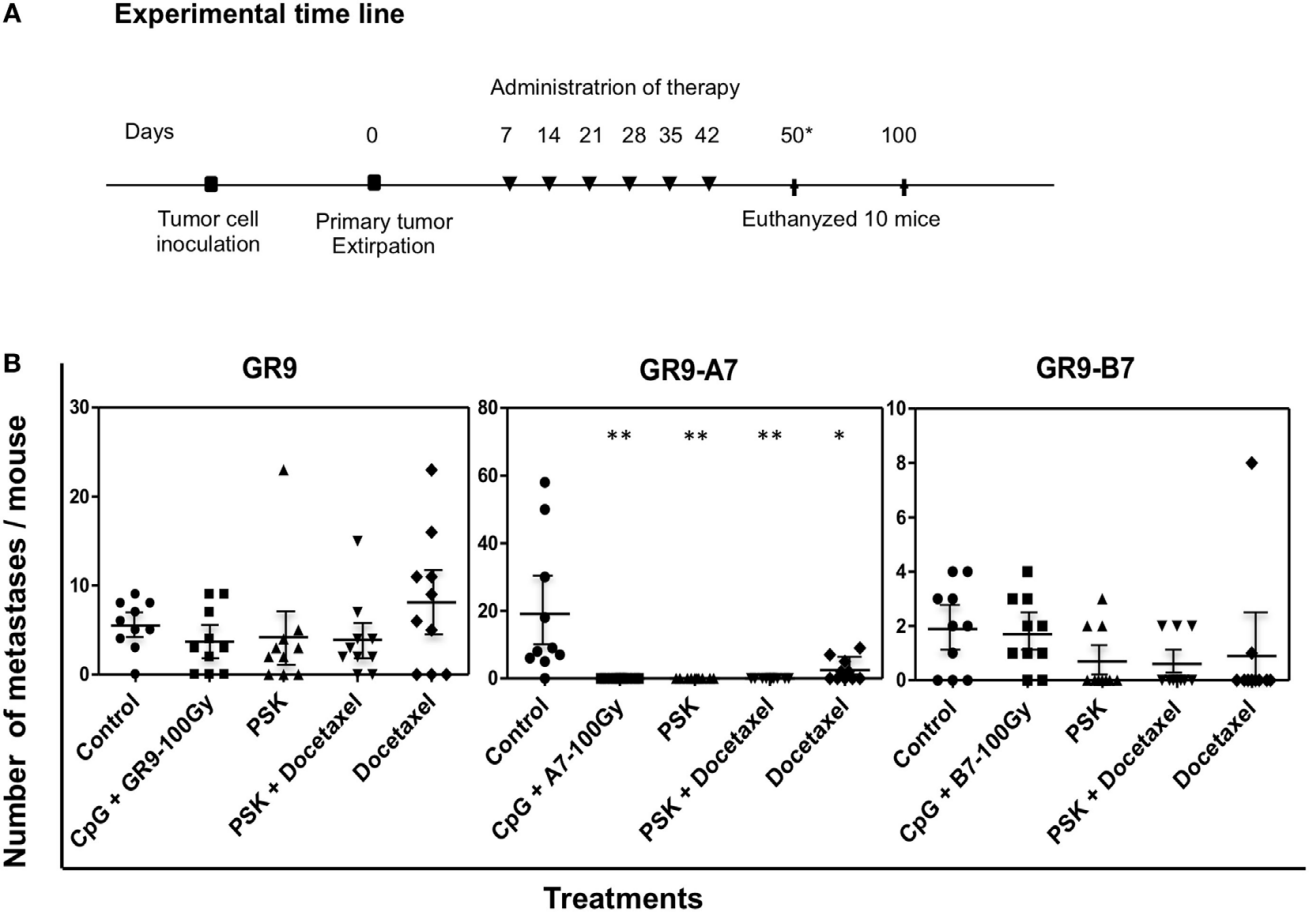
**(A)** Treatment schedules. Treatments were administered weekly for six consecutive weeks by intraperitoneal injection, beginning at 1 week after removal of the primary tumor. Control groups injected with tumor cells (GR9 or GR9-A7 or GR9-B7) received saline solution as treatment. Ten mice were sacrificed on day 50 post-tumor extirpation to analyze the treatment efficacy. The remaining 10 mice in each group were sacrificed on day100 post-tumor extirpation to evaluate the long-term effect. *Mice in the control and docetaxel groups were sacrificed on day 50 post-tumor extirpation. **(B)** Spontaneous metastases in tumor-bearing mice receiving the different therapies. The figure shows the pulmonary metastasis count per mouse. These results were reproduced in another two independent experiments. *p < 0.05; **p < 0.01 vs. control. Kruskal–Wallis test, followed by Dunn *post hoc* test was used for statistical analysis.

GR9, GR9-A7, and GR9-B7 were, therefore, selected as candidates to evaluate the response to anti-metastatic therapy. Injected cell doses were 12.5 × 10^5^ cells for GR9 and the A7 clone and 25 × 10^5^ cells for the B7 clone, i.e., doses at which these tumor cells exhibit high spontaneous metastatic capacity. Figure 4A depicts administration protocol of the four performed treatments (see Materials and Methods). The therapeutic strategies were as follows: (a) CpG+ irradiated cells for activation of tumor antigen presentation and of the T-cell immune response, focused on the recognition of tumor cells expressing tumor antigens; (b) PSK for activation of NK cells to recognize MHC-I low tumor cells (PSK may also increase T-cell subsets); (c) docetaxel for the comparison of chemotherapy with immunotherapy; and (d) PSK + docetaxel for comparison with the effects of PSK immunotherapy and docetaxel chemotherapy alone.

In the GR9-A7 group, metastases were detected in 9 of the 10 untreated mice (range 6–58 PMs; range 0–4 LNMs) and in 5 of those treated with docetaxel (2–9 PMs; 0–6 LNMs) (Figure 4B). By contrast, all of the 10 GR9-A7 mice treated with CpG+IR−TC, PSK, or PSK plus docetaxel were free of overt metastases at 50 days post-tumor extirpation (Figure 4B), and the other 10 mice in these groups were also metastasis-free after euthanasia on day 100 post-tumor extirpation (50 days after their last treatment). The GR9-B7 clone produced PMs in 7 of the 10 mice in the untreated group (range 1–4), whereas PMs were observed in only 3 of the 10 mice treated with PSK (2–3 PMs; 0–1 LNMs), 3 of the 10 mice treated with PSK plus docetaxel (2 PMs), and 2 of the 10 mice treated with docetaxel (range 1–8 PMs); however, metastases were detected in 8 out of the 10 mice treated with CpG + IR−TC (1–4 PMs; 0–1 LMNs), similar to the prevalence in untreated animals (Figure 4B). In comparison to the untreated GR9 group, the percentage of animals with metastases was only slightly reduced from 90 to 70% by treatment with CpG + IR−TC, docetaxel, or PSK and to 80% by treatment with PSK + docetaxel. The mean metastasis count per mouse was lower in the mice receiving immunotherapy or chemoimmunotherapy but higher in those treated with docetaxel (Figure 4B).

### Changes in Immune Cell Subpopulations Generated in the Mice by the Different Treatments

Host immune cell populations were determined 50 days after tumor primary extirpation, at 1 week after the end of the treatment. In the GR9-A7 groups, immunotherapy and chemoimmunotherapy each produced a significant (*p* < 0.05) increase in TCD3+ lymphocytes (46.5, 58.0, and 36.6 vs. 30.4%) and in CD4+ (31.9, 44.2, and 30.1 vs. 24.4%) and CD8+ (14, 13.7, and 6.5 vs. 5.8%) T-cell subsets in comparison to untreated tumor-bearing mice, with a decrease in Treg cells (4.7, 9.5, and 1.1 vs. 10.8%) (Table 1). Immunotherapy and chemoimmunotherapy protocols were completely effective to reverse the alterations in immune cell subsets promoted by the GR9-A7 tumor clone and abrogate the metastatic colonization.

In GR9-B7 tumor-bearing mice, treatments with PSK and PSK plus docetaxel produced a slight recovery in TCD3+ (32.2 and 26.0 vs. 25.3%, respectively) due to an increase in CD4+ lymphocytes (23.0 and 20.6% vs. 19.7%) vs. untreated tumor-bearing mice, but a significant increase in Treg cells (9.0 and 9.6 vs. 6.9%) was also observed (Table 1). Hence, the immune status was not completely reversed with immunotherapy and chemoimmunotherapy treatments of GR9-B7 groups, with only a partial decrease in metastasis colonization. In contrast, treatment with CpG+IR−TC was completely ineffective. In the GR9 tumor-bearing mouse group, immunotherapy with PSK and chemoimmunotherapy with PSK + docetaxel partially reversed the alterations in immune cell subsets generated by GR9 tumor cells, producing a significant increase in CD3+ T lymphocytes (32.1 and 30.8 vs. 18.0%, respectively) due to an increase in TCD4+ (26.0 and 19.7 vs. 14.6%) and TCD8+ (6.2 and 8.5 vs. 3.4%) lymphocytes. In brief, none of the treatments was able to completely reverse the alterations in immune cell subsets in GR9 and GR9-B7 mice groups, unlike in the GR9-A7 group. Analysis of lymphocyte subpopulations in peripheral blood yielded similar results (data not shown).

### Generation of a New Diversity in MHC-I Expression on Metastases

**Figure 5:**
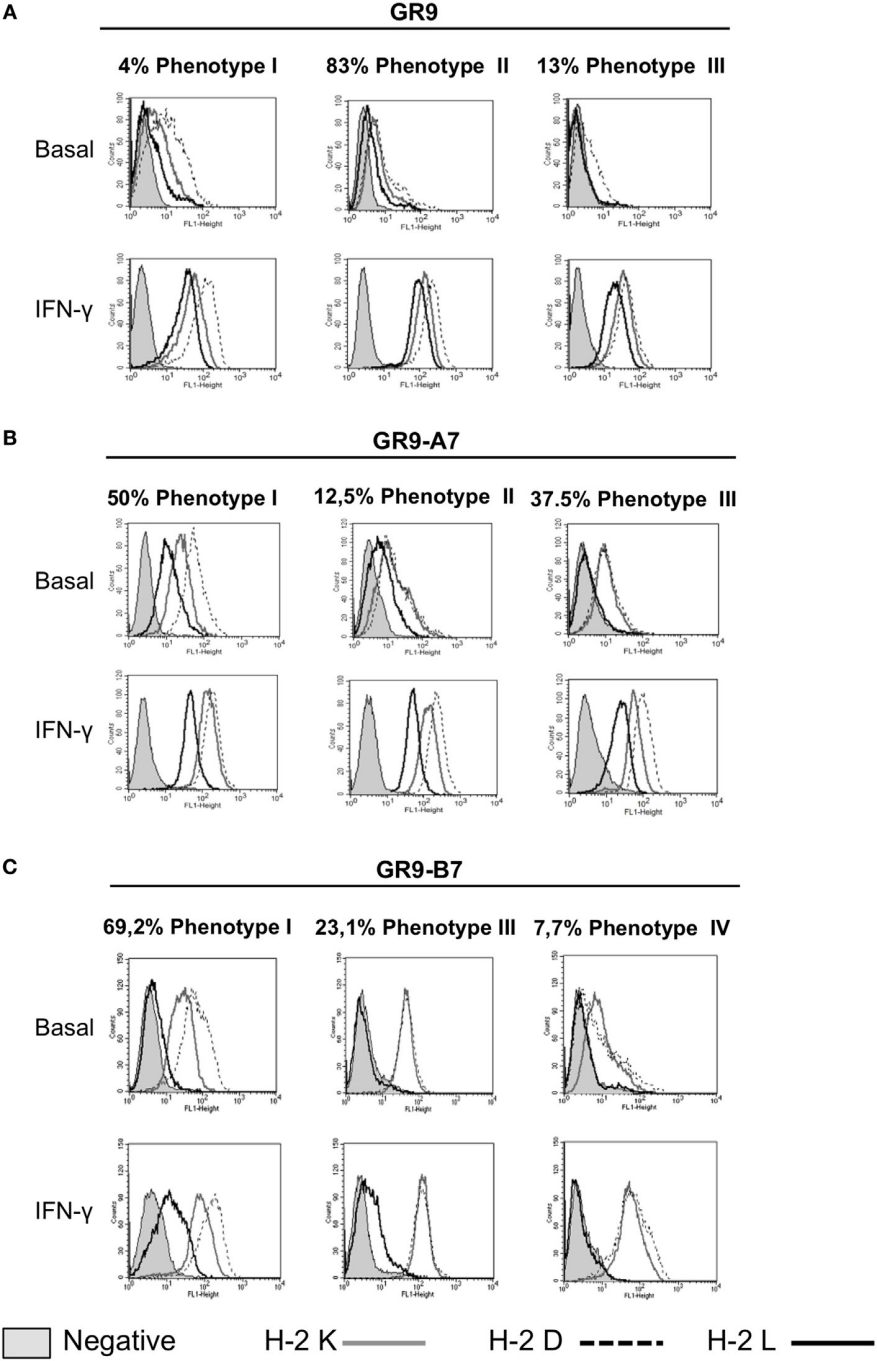
MHC-I phenotypes of GR9, GR9-A7, and GR9-B7 spontaneous metastases under baseline conditions and after treatment with IFN-γ: H-2 Kd (*gray line), H-2 Dd (dotted line), and H-2* Ld (*black line)*. Four different MHC-I phenotypes were found. **(A)** MHC-I phenotypes of GR9 spontaneous metastases; **(B)** MHC-I phenotypes of GR9-A7 spontaneous metastases; **(C)** MHC-I phenotypes of GR9-B7 spontaneous metastases. Data from a representative experiment are depicted.

The spontaneous metastases derived from GR9, GR9-A7, GR9-B7, and GR9-C5 exhibited a second heterogeneity in MHC-I cell surface expression. Metastases were classified into four types according to the relationship of their H-2 surface expression with that of the corresponding tumor clone: Phenotype I, with similar H-2 expression to that of the injected tumor cells; Phenotype II, with reversible downregulation of all three H-2 molecules; Phenotype III, with partial downregulation of all three H-2 molecules and total and reversible loss of H-2 D^d^ and/or H-2 L^d^ molecules; and Phenotype IV, with partial and reversible downregulation of H-2 K^d^ and D^d^ molecules and total irreversible loss of H-2 L^d^ allele. Reversible or irreversible defects were defined by the induction or non-induction of surface expression of each H-2 allele after IFN-γ treatment. Phenotype I was recorded for 4% of GR9-derived spontaneous metastases, phenotype II for 83%, and phenotype III for 13% (Figure 5A). Phenotype I was recorded for 50% of spontaneous metastases derived from the GR9-A7 tumor cell clone, phenotype II for 12.5%, and phenotype III for 37.5% (Figure 5B). Among metastases derived from GR9-B7 cell clone, 69.2% were classified as phenotype I, 23.1% as phenotype III, and 7.7% as phenotype IV (Figure 5C). The H-2 class I expression of all metastases from GR9-C5 clone was the same as that of the original clone (data not shown). MHC-I expression was highest in metastases derived from GR9-A7 clone, followed by metastases from GR9-B7 clone, and was lowest in those derived from GR9 tumor cells.

### Experimental Metastatic Potential of These Tumor Cell Clones

The experimental metastasis assay protocol completely differed from that of the spontaneous metastasis assays, directly injecting tumor cells into the blood stream and thereby excluding initial stages of local tumor growth and extravasation. GR9-A7 and B11 clones were selected for experimental metastatic assay because of their differences in H-2 class I expression. At day 30 post-cell injection, necropsy showed that 100% of mice injected with GR9-A7 cells were metastasis-free, whereas 80% of mice injected with GR9-B11 presented overt metastases (Table 2). These findings are the opposite of the spontaneous metastasis results.

**Table 2:** Experimental metastasis assays with GR9-A7 and GR9-B11 tumor cell clones.

Cell lines	Cell dose	Number of mice	Mice with metastases	Number of metastases	
				Micro-PMs^a^	Macro-PMs^b^
GR9-A7	2.5 × 10^5^	10	0/10	0	0
GR9-B11	2.5 × 10^5^	10	8/10	2–6	1–4

*^a^Micro-PMs: pulmonary micrometastases*.

*^b^Macro-PMs: pulmonary macrometastases*.

## Discussion

**Figure 6:**
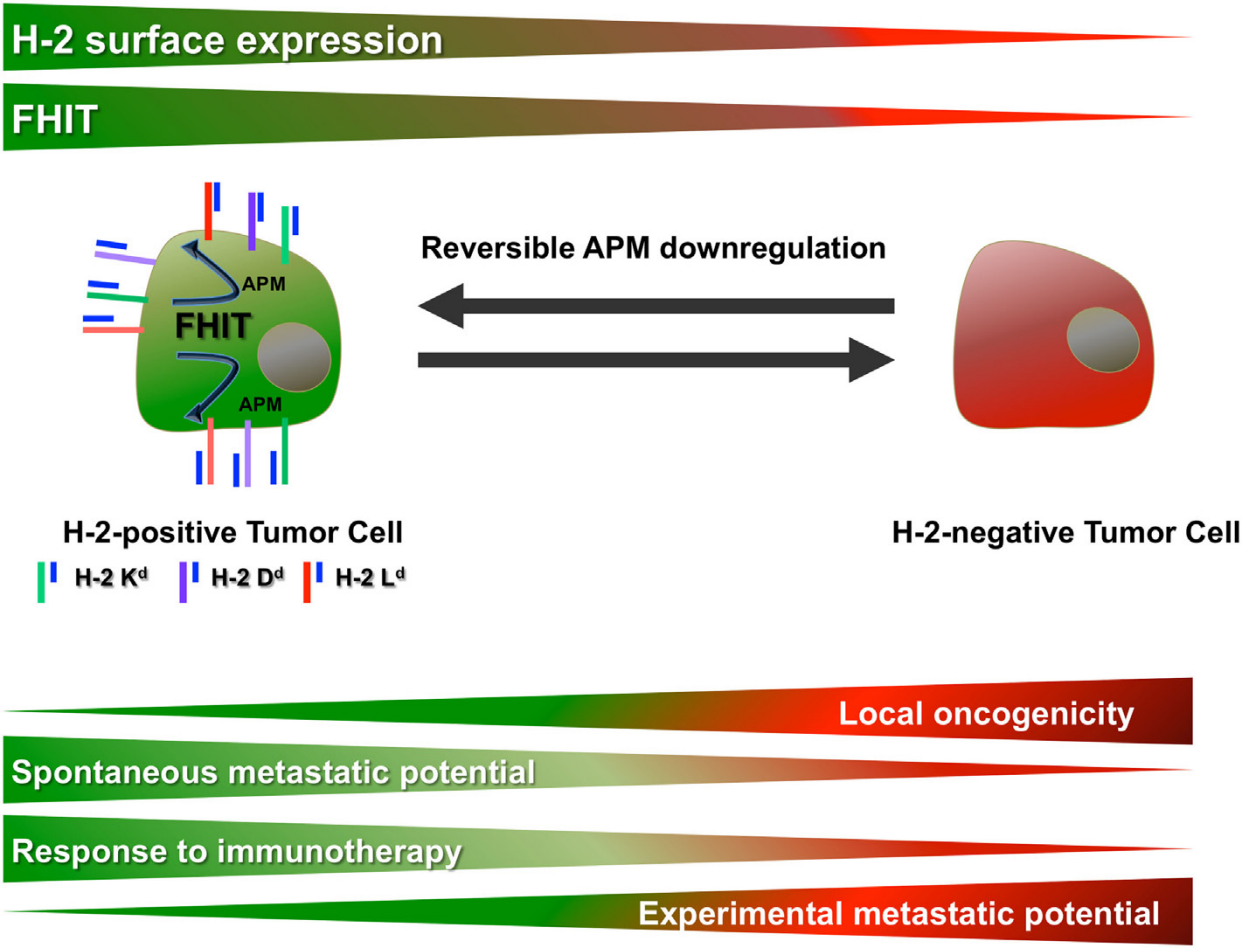
Influence of MHC-I and Fhit expression in primary tumors on their oncogenic and metastatic potential and response to immunotherapy.

Intratumoral genetic heterogeneity has been widely reported in cancer and can determine the biological characteristics and progression of tumors (18–20). However, reversible phenotypic intratumoral heterogeneity may also be important (21, 22). The present results indicate that intratumoral heterogeneity in the MHC-I cell surface expression (MHC-I phenotype) of a tumor may determine its oncogenic potential, metastatic capacity, and response to immunotherapy (Figure 6). Cell clones isolated from a MCA-induced primary fibrosarcoma in BALB/c mice had a wide range of MHC-I phenotypes, from negative to highly positive. MHC-I downregulation was observed in some clones but was reversible after IFN-γ treatment (soft MHC-I lesions). Coordinated transcriptional downregulation of APM genes is responsible for these alterations in MHC-I surface expression. Our group previously reported that loss of expression of the Fhit tumor suppressor gene may be responsible for the transcriptional downregulation of APM components (16), and the present results show the loss of Fhit transcriptional expression in clones with low MHC-I phenotype. Alterations in MHC-I surface expression are attributable to epigenetic mechanisms, given that expression was restored after TSA treatment.

Examination of the *in vivo* biological behavior of the cell clones revealed an inverse correlation between MHC-I phenotype and local oncogenicity, finding a higher local tumor growth rate for clones with lower MHC-I expression (Figure 6) (23). In addition, Fhit gene transfection of the MHC-I-negative GR9-B11 clone completely reversed the MHC-I downregulation, transforming it into a highly immunogenic and non-oncogenic clone and this immunorejection was mediated by CD8+ T lymphocytes (manuscript in preparation). We previously reported that total loss of HLA expression generated more oncogenic tumor cells in human tumors and that transfection of one lost HLA allele reverted these oncogenic properties, showing that MHC-I molecules may act as tumor suppressor genes (8, 11). Taken together, these results indicate that the loss of MHC-I expression or of genes co-regulated with MHC expression may contribute to increased local oncogenicity. By contrast, the *in vivo* spontaneous metastatic potential was greater in clones with high MHC-I expression than in those with low MHC-I expression, showing a direct correlation with the MHC-I profile (Figure 6). Hence, the spontaneous metastatic capacity cannot always be extrapolated from the local oncogenicity or *vice-versa*, and they may change in opposite directions. According to this finding, the approach to a localized primary tumor may not be appropriate for advanced metastatic disease.

Analysis of the immune cell populations in tumor-bearing mice showed that the highly metastatic (MHC-I positive) tumor cell clones induced a strong decrease in several immune cell subsets in the host. Given that these clones and their metastases are MHC-I positive, this decrease might be attributable to an immune escape mechanism. The same may occur in patients with MHC-I positive tumors, allowing tumor cells to progress and avoid their destruction. If this decrease can be reversed, their MHC-I positive metastases might be recognized by the immune system. Thus, GR9-A7 and their metastases showed the highest MHC-I positivity, but immunotherapy completely reversed the decrease in immune subpopulations and halted metastatic dissemination in GR9-A7 tumor-bearing mice (24). By contrast, immunotherapy partially reverted the decrease in immune cell subsets and also partially reverted metastatic dissemination in mice bearing tumors from the GR9-B7 tumor clone, which showed intermediate MHC-I expression, as did its metastases. A strong decrease in T lymphocytes and a strong increase in Treg cells were observed in mice bearing tumors from GR9 tumor cells, which include all four tumor cell clones, and the overt metastases detected had the lowest MHC-I surface expression. In these animals, the immunotherapy did not reverse these changes in immune subpopulations or halt metastatic progression. In the mice bearing tumors from the MHC-I negative GR9-B11 tumor cell clone, a strong immunostimulation was produced, with no detection of overt metastases during follow-up; however, overt metastases appeared after these animals were depleted of CD8+ T lymphocytes, indicating that the immune response mediated by CD8+ T lymphocytes kept the metastases in a permanent dormant state (17). Interestingly, these dormant metastases presented a MHC-I positive phenotype. According to these findings, the metastasis prognosis is worse for highly MHC-I positive tumor cell clones, but they may respond very well to immunotherapy, to which tumors with intermediate MHC-I phenotype do not fully respond. The metastatic prognosis may be good for MHC-I negative tumor clones in some cases, producing MHC-I positive metastases in an immunodormancy state mediated by TCD8+ lymphocytes (Figure 6). In brief, the MHC-I phenotype of cancer cells and the host immune status, especially in relation to T lymphocytes, may determine the response of metastases to immunotherapy. It may, therefore, be of interest to diagnose the MHC-I phenotype of cancer cells and the T-cell subsets of cancer patients before ordering immunotherapy (25). Patients with MHC-I positive tumors or metastases who show low T-cell subsets may be good candidates for immunotherapy (26, 27). Likewise, in certain types of human cancer, absent or positive MHC-I expression on primary tumor cells is a marker of good prognosis, whereas a moderate downregulation of MHC-I expression is not. Thus, the highly positive expression of MHC-I molecules or their total loss have been associated with a better prognosis in colorectal tumors (28, 29) while downregulation of APM component and HLA-I expression has been associated with shorter recurrence free and overall survival in cervical cancer and triple-negative breast cancer patients (30, 31). Recent studies revealed that detection of HLA class I and CD8^+^ T-cell infiltration is a good prognostic marker of the clinical outcome in ovarian cancer and biliary tract cancer (26, 32). Total loss of MHC-I expression was found to be an independent indicator of a good prognosis in breast cancer (33), and HLA class I and Treg levels were reported to predict the response to chemotherapy in early breast cancer (27).

During the metastatic process, a new heterogeneity in MHC-I phenotypes was observed, finding differences in MHC-I phenotype between the overt metastases and the tumor clone from which they derived. In all cases, a downregulation in MHC-I phenotype was observed in >45% of overt metastases. In the case of GR9 tumor cells, 96% of the metastases showed a decrease in MHC-I phenotype, which might explain the failure of immunotherapy in these mice. By contrast, metastases from the GR9-A7 tumor clone had a high MHC-I phenotype, and the immunotherapy was completely effective. The metastases from GR9-B7 had an intermediate MHC-I positive phenotype, and the immunotherapy was partially effective. It, therefore, appears that the MHC-I phenotype of metastases might determine the effectiveness of immunotherapy. Activation of the immune system by immunotherapy might be effective and enduring if immune cells are able to recognize MHC-I positive metastatic cells. The downregulation or selective loss of HLA-I expression vs. the primary tumor has been observed in LNM from humans with breast, colorectal, gastric, laryngeal, cervical, or head and neck cancers, suggesting an association between the loss of HLA-I molecules and lymph node metastasis (34–38). Loss of MHC-I cell surface expression has also been described in progressive metastases from melanoma after immunotherapy (39), and MHC-I downregulation in cancer cells was recently reported to be a driver of resistance to chemotherapy and immunotherapy in patients with melanoma or prostate cancer (40–42).

Interestingly, none of the tumor cell clones under study had *in vivo* spontaneous metastatic potential when 5 million cells were injected, including clones showing high spontaneous metastatic potential at lower cell doses. The size of all excised primary tumors was the same (largest diameter of 10 mm), regardless of the cell dose, indicating that metastatic dissemination is influenced not only by tumor size but also by the time period of local tumor growth. In the present study, metastatic colonization was achieved at tumor cell doses, when the growth of primary tumors was slower. The role of early-disseminated tumor cells in metastatic progression at a secondary site is a controversial issue (43–47). Our data suggest that GR9 tumor cell clones cannot initiate metastatic colonization at secondary sites over the short term.

A striking finding in our cancer model was the inverse correlation between spontaneous and experimental metastatic potential, with higher experimental metastatic capacity being found for the clones with lower MHC-I expression. In other words, one tumor cell clone may present high spontaneous metastatic potential and null experimental metastatic potential and another may present null spontaneous metastatic potential and high experimental metastatic potential. Spontaneous metastasis assays faithfully resemble metastatic process in human cancers from primary local tumor to secondary colonization (48, 49). However, primary tumor cells are injected directly into the bloodstream in experimental metastasis assays, so that not all of the sequential steps in the metastatic cascade are reproduced. Experimental metastatic assays are frequently used to evaluate the metastatic potential of a primary tumor and the effectiveness of anti-metastatic treatments. However, our findings suggest that these assays may fail to reproduce the metastatic advance of the disease and the response to pharmacological therapies in preclinical animal models.

In summary, an individual cancer should be considered as heterogeneous, comprising different tumor and metastasis cell clones that may determine the metastatic progression, antitumor immunosurveillance, and response to immunotherapy. The development of precise tumor targeting strategies, therefore, requires elucidation of the tumor and metastatic subpopulations of a given cancer. Our proposed tumor model represent a first step in understanding how the presence of different MHC-I phenotypes of cell clones in the primary tumor can influence the success or failure of antitumor treatments. Levels of MHC-I expression on cancer cells may modulate the oncogenicity, metastatic progression, and response to immunotherapy. The present results suggest that a high MHC-I phenotype of the primary tumor and metastases predict a good anti-metastatic response to immunotherapy. The MHC-I phenotype on primary tumors and metastases and the T immune cell subsets of the patient may serve as predictive markers of both the prognosis and the response to immunotherapy.

## Ethics Statement

This study was carried out in accordance with the recommendations of European Community Directive 86/609/CEE and Spanish law (Real Decreto 1201/2005) on the use of laboratory animals, and their housing and the experimental procedures were approved by the Junta de Andalucía animal care committee and adhered to animal welfare guidelines of the National Committee for Animal Experiments.

## Author Contributions

IR, FG, and AG-L: design and supervision of the study, and writing and revising the manuscript. IR, CG, IA, VC, AC, and AG-L: development of methodology, acquisition of data and analysis, and interpretation of data. All authors revised the manuscript, approved the final version, and agreed to be accountable for the work.

## Conflict of Interest Statement

The research was conducted in the absence of any commercial or financial relationships that could be construed as a potential conflict of interest.
